# How the Human Brain Detects Unexpected Events

**DOI:** 10.1371/journal.pbio.0040443

**Published:** 2006-11-28

**Authors:** Liza Gross

We’ve all had the experience of walking into a place we know well and immediately and apparently effortlessly noticing that something has changed—for example, the photo of your mother-in-law, once discretely displayed on a corner table, now occupies a prominent position on the mantle. Although neuroscientists have spent over 40 years investigating how the brain reacts to novel stimuli in the environment, they are only just beginning to understand how the brain detects “associative novelty,” where familiar objects appear in new configurations.

Computational models strongly suggest that the hippocampus plays a crucial role in novelty detection by perceiving disparities between expectations based on past experience and sensory reality. These models propose that the hippocampal CA1 region compares prior predictions emanating from the CA3 region with sensory inputs arriving from the nearby entorhinal cortex. “Associative mismatches,” therefore, result when current sensory inputs are at odds with what would be predicted based on stored representations. By this account, we only notice that the photo has moved because we have expectations, derived from past experience, about where it should be located. Until now, however, empirical evidence that the human hippocampus operates in this way has been lacking.

In a new study, Dharshan Kumaran and Eleanor Maguire used functional magnetic resonance imaging (fMRI) and behavioral experiments to explore the processing of sequence novelty (that is, temporal associative novelty) in humans. The authors examined brain responses to novel sequences of objects while participants performed an incidental target-detection task designed to emphasize the automatic processing of novelty. Participants first saw four novel objects presented in consecutive order, followed after a brief delay, by the presentation of the same objects in one of three orders: the same, half-new (first two objects in the same order and the last two in reverse order), or entirely new. The authors used nearly 1,000 images, so that individuals saw each object only twice during the experiment (once during the first and second presentations).

This experimental design allowed the authors to identify whether novelty-related responses reflected the operation of comparison-based associative match-mismatch computations, or alternatively, a more general response to sequence novelty per se. Critically, an associative mismatch situation only arises in the half-new condition, where predictions derived from prior experience (that is, the first presentation), and cues by the first two objects in the sequence are violated.

**Figure:**
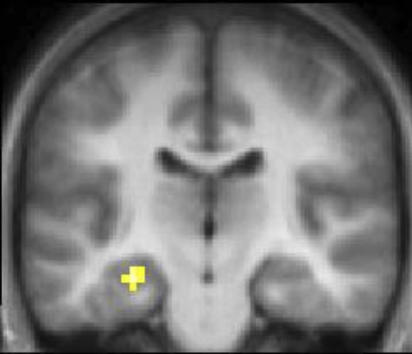
Hippocampal activity signals the presence of a mismatch between what is expected to happen and what actually does.

The authors looked for differences in brain activity between the three possible sequential orders in which objects could appear during the second presentation. Although hippocampal activity was initially indistinguishable for the repeated and half-new sequence order, it increased substantially in the half-new situation when the third object appeared and was different from the one expected. Thus, the hippocampus was maximally engaged when reality ran counter to prior expectations about what would happen next, and not in response to sequence novelty per se. Moreover, this violation of expectations had a striking effect on subjects’ behavior: in a separate experiment, participants’ reaction times markedly slowed in the presence of associative mismatch in the half-new condition.

This study provides direct evidence that the hippocampus acts as an associative match-mismatch detector (or comparator device) and responds, not to novelty per se, but to discrepancies between expected and received signals in the environment. In contrast, the entorhinal cortex, a region with strong reciprocal connections with the hippocampus, exhibited a different pattern of neural activation consistent with a more general response to sequence novelty. These findings also provide empirical support for the view that the hippocampus plays a critical role in storing representations of event sequences and, specifically, in replaying entire stored sequences in response to a partial input cue (the first object in the sequence). And it may be this unique capability that underlies the essential role of the hippocampus in recalling the details of an event, and perhaps even the episodic vignettes that tell the story of one’s life.

